# Assessing the impact of natural policy experiments on socioeconomic inequalities in health: how to apply commonly used quantitative analytical methods?

**DOI:** 10.1186/s12874-017-0317-5

**Published:** 2017-04-20

**Authors:** Yannan Hu, Frank J. van Lenthe, Rasmus Hoffmann, Karen van Hedel, Johan P. Mackenbach

**Affiliations:** 1 Erasmus MC, P.O. Box 2040, Rotterdam, 3000 CA The Netherlands; 2 European University Institute, Florence, Italy; 3 Max Planck Institute for Demographic Research, Rostock, Germany

## Abstract

**Background:**

The scientific evidence-base for policies to tackle health inequalities is limited. Natural policy experiments (NPE) have drawn increasing attention as a means to evaluating the effects of policies on health. Several analytical methods can be used to evaluate the outcomes of NPEs in terms of average population health, but it is unclear whether they can also be used to assess the outcomes of NPEs in terms of health inequalities. The aim of this study therefore was to assess whether, and to demonstrate how, a number of commonly used analytical methods for the evaluation of NPEs can be applied to quantify the effect of policies on health inequalities.

**Methods:**

We identified seven quantitative analytical methods for the evaluation of NPEs: regression adjustment, propensity score matching, difference-in-differences analysis, fixed effects analysis, instrumental variable analysis, regression discontinuity and interrupted time-series. We assessed whether these methods can be used to quantify the effect of policies on the magnitude of health inequalities either by conducting a stratified analysis or by including an interaction term, and illustrated both approaches in a fictitious numerical example.

**Results:**

All seven methods can be used to quantify the equity impact of policies on absolute and relative inequalities in health by conducting an analysis stratified by socioeconomic position, and all but one (propensity score matching) can be used to quantify equity impacts by inclusion of an interaction term between socioeconomic position and policy exposure.

**Conclusion:**

Methods commonly used in economics and econometrics for the evaluation of NPEs can also be applied to assess the equity impact of policies, and our illustrations provide guidance on how to do this appropriately. The low external validity of results from instrumental variable analysis and regression discontinuity makes these methods less desirable for assessing policy effects on population-level health inequalities. Increased use of the methods in social epidemiology will help to build an evidence base to support policy making in the area of health inequalities.

## Background

There is overwhelming evidence for the existence of socioeconomic inequalities in health in many countries [1–3]. Improvements in understanding their underlying mechanisms have reached a point where several entry-points have been identified for interventions and policies aimed at reducing health inequalities [2, 4]. The latter has often been made a priority in national and local health policy [2, 5–9]. Yet, the scientific evidence-base for interventions and policies to tackle health inequalities is still very limited, and mostly applies to the proximal determinants of health inequalities such as smoking and working conditions [10–14]. Policies that address the social and economic conditions in which people live probably have the greatest potential to reduce health inequalities, but these are the hardest to evaluate [15].

Randomized controlled trials (RCTs) are regarded as the “gold standard” in the effect evaluation of clinical studies. The limitations of RCT’s in evaluating policies in public health, however, have been clearly recognized [16, 17]. For policies aimed at tackling health inequalities, an obvious limitation is that policies to improve material and psychosocial living conditions, access to essential (health care) services, and health-related behaviors often cannot be randomized.

Natural policy experiments (NPEs), defined as “policies that are not under the control of the researchers, but which are amenable to research using the variation in exposure that they generate to analyze their impact” have been advocated as a promising alternative [18, 19]. In NPEs, researchers exploit the fact that often not all (groups of) individuals are exposed to the policy, e.g. because some individuals are purposefully assigned to the policy and others are not, or because the policy is implemented in some geographical units but not in others. For example, a policy to improve housing conditions in neighborhoods might be implemented in neighborhoods where the need to do so is largest, or some cities may decide to implement the policy and others not. Of course, in these cases those in the intervention and control group are likely to differ in many other factors than exposure to the policy, and analytical methods will have to adequately control for confounding in order to allow reliable causal inference.

The application of methods for the evaluation of NPEs, such as difference-in-differences and regression discontinuity, is reasonably well advanced in economics and econometrics. While these methods have also entered the field of public health [20, 21], and have been applied occasionally to study policy impacts on health inequalities [22, 23], there is as yet no general understanding of whether and how each of these methods can be applied to assess the impact of policies on the magnitude of socioeconomic inequalities in health. If they can, however, they can help to extend the evidence-base in this area substantially.

The main aim of this study therefore is to assess whether, and to demonstrate how, a number of commonly used analytical methods for the evaluation of NPEs can be applied to quantify the impact of policies on health inequalities. In doing so, we will also pay attention to two issues that may complicate assessing the impact of policies on socioeconomic inequalities in health. Firstly, socioeconomic inequalities in health can be measured in different ways. Secondly, policies may reduce health inequalities in different ways.

With regard to the measurement of health inequalities, it is important to distinguish relative and absolute inequalities. Relative inequalities in health are usually measured by taking the ratio of the morbidity or mortality rate in lower socioeconomic groups relative to those in higher socioeconomic groups, e.g. an odds ratio (OR), a rate ratio (RR), or a relative index of inequality [24]. Absolute inequalities in health are usually measured by taking the difference between the morbidity or mortality rates of lower and higher socioeconomic groups, e.g. a simple rate difference or the more complex slope index of inequality [24]. Relative and absolute inequalities both are considered important, although it is sometimes argued that a reduction in absolute inequalities is a more relevant policy outcome than a reduction in relative inequalities, because it is the absolute excess morbidity or mortality in lower socioeconomic groups that ultimately matters most for individuals. Nevertheless, quantitative methods used for the evaluation of policies should be able to measure the impact on both absolute and relative inequalities in health.

With regard to the second issue, there are two ways through which a policy can reduce socioeconomic inequalities in health: (1) the policy has a larger effect on exposed people in lower socioeconomic group, or (2) more people in lower socioeconomic group are exposed to it. Clearly, both can also occur simultaneously; raising the tax on tobacco may affect individuals with lower incomes more than those with higher incomes, and given the higher prevalence of smokers in low income groups also affects more smokers in low than high income groups. In fact, changes in aggregated health outcomes collected for a country or region (e.g. mortality rates or the prevalence of self-assessed health) after the introduction of a policy are the result of an effect among the exposed as well as the proportion of exposed persons. For the ultimate goal to assess whether a reduction in health inequalities in the population occurred this is less relevant – one could argue that eventually only the end result counts, that is a change in the magnitude of socioeconomic inequalities in health. Many statistical techniques, however, ‘only’ provide the effect of the policy among the exposed; they do not take into account the proportion of persons exposed to a policy. In order to be able to quantify the impact of a policy on socioeconomic inequalities in health in a population, an additional step is then needed: the policy effect should be combined with information about the proportion of exposed persons in higher and lower socioeconomic groups.

The structure of this paper is as follows. We first describe a fictitious data example that allowed us to assess the applicability of seven commonly used analytic methods techniques for evaluating NPEs, which we also briefly describe. We then demonstrate the use of these methods for assessing the impacts of policies on the magnitude of health inequalities in our fictitious dataset. Finally, we discuss the advantages and disadvantages of the various methods.

## Methods

### A fictitious data example

We generated a fictitious dataset of 20,000 residents of a city. In this city, half of the residents were regarded as in lower educational group, and within each educational group there were 50% males. The health outcome that we used was self-assessed health, dichotomized into either ‘poor’ or ‘good’. The numbers (shown in Table 1) were chosen such that the proportion of persons with poor health before the introduction of the policy was higher among the lower educational group (20%) than among the higher educational group (10%). In order to make gender as a confounder, we constructed the data such, that women had better health (10% with poor health) than men (20% with poor health). At one point in time, the city council introduced a free medical care service in a number of neighborhoods, most of which were deprived. Thus, relatively more people in lower educational group were exposed to the policy (50%) as compared to people in higher educational group (25%). At the same time, more women (75% in lower educational group and 37.5% in higher educational group) than men (25% in lower educational group and 12.5% in higher educational group) used the free health care within each educational group. Because women had better health before the introduction of the policy and tended to be more exposed to the intervention, gender was a confounder in the association between the policy exposure and self-assessed health.

**Table 1:** Numbers of residents in a city: a fictitious dataset

Education (n)	Sex (n)	Policy allocation (n)	Self-assessed health	Before the policy (Health t_1,_%)	After the policy (Health t_2_, %)
Low (10000)	Male (5000)	Exposed^a^ (1250)	Poor	333 (27%)	221 (18%)
			Good	917 (73%)	1029 (82%)
		Unexposed (3750)	Poor	1000 (27%)	950 (25%)
			Good	2750 (73%)	2800 (75%)
	Female (5000)	Exposed (3750)	Poor	500 (13%)	333 (9%)
			Good	3250 (87%)	3417 (91%)
		Unexposed (1250)	Poor	167 (13%)	159 (13%)
			Good	1083 (87%)	1091 (87%)
High (10000)	Male (5000)	Exposed (625)	Poor	83 (13%)	46 (7%)
			Good	542 (87%)	579 (93%)
		Unexposed (4375)	Poor	584 (13%)	467 (11%)
			Good	3791 (87%)	3908 (89%)
	Female (5000)	Exposed (1875)	Poor	125 (7%)	70 (4%)
			Good	1750 (93%)	1805 (96%)
		Unexposed (3125)	Poor	208 (7%)	166 (5%)
			Good	2917 (93%)	2959 (95%)

^a^exposure was defined as actually using the free medical care service

We assumed that the effect of the policy was a reduction of the prevalence (or probability) of poor health among the exposed of 30%, regardless of their education level. Moreover, we imposed a naturally occurring recovery from poor to good health: even without the intervention, people in higher educational group had a 20% chance of reverting to good health and people in lower educational group had a 5% chance of reverting to good health. This could be due to spontaneous recovery or to external conditions such as other policies or changes in macroeconomic factors, which were not directly related to the policy introduced. As a result, and for example, the number of men with lower education who had poor health and who were exposed to the policy declined from 333 before the policy was implemented to 221 (333*0.70*0.95) after the policy was implemented (see Table 1). As those with good health were assumed not to change to poor health, the number of men in lower educational group exposed to the policy with good health became 1029 (917 + (333–221)). Similarly, and as another example, the number of women in higher educational group unexposed to the policy with good health after the introduction of the policy became 2959 (2917 + 208*0.2). We assumed that health could only change from poor to good, in order to make the fictitious dataset simpler. In reality, health can also deteriorate over time. Allowing the deterioration in health will not change the feasibility of all the listed methods and the way of implementing the methods.

Compared to men, a smaller proportion of women reported poor health before the policy, and more women were exposed to the policy: the proportion of poor health before the policy was 20% (2000/10,000) among men and 10% (1000/10,000) among women, and the proportion of persons exposed to the policy was 56.25% for women (5625/10,000) and only 18.75% for men (1875/10,000). Gender thus was a confounder of the relation between policy exposure and health.

### Quantitative methods for the evaluation of natural policy experiments

To identify potentially relevant quantitative methods for the evaluation of NPEs, we started by reviewing the classical econometric literature [20, 25–31]. Seven quantitative methods were identified as potentially suitable for the evaluation of NPE’s (Table 2): (1) regression adjustment, (2) propensity score matching, (3) difference-in-differences analysis, (4) fixed effects analysis, (5) instrumental variable analysis, (6) regression discontinuity and (7) interrupted time-series. We will not elaborate upon the general application of these methods – for this we refer the reader to existing textbooks and papers [20, 25, 31, 32]. Nevertheless, a basic understanding of the concepts behind these techniques is important for our purposes.

**Table 2:** Concepts, limitations and applications of statistical approaches for the evaluation of natural policy experiments

Method	Main concept	Minimum data requirement	Adjustment for confounders	Main limitations	Application to the evaluation of policies on health inequalities
Regression adjustment	Adjustment for confounders, i.e. factors related to both intervention allocation and health outcomes.	Cross-sectional	Observed confounders	Vulnerable to unobserved confounders	[50]
Propensity score matching	For a given propensity score, exposure to the intervention is random. The intervention effect is therefore the average difference in the outcomes between the exposed and the matched unexposed units with the same propensity scores.	Cross-sectional	Observed confounders	Vulnerable to unobserved confounders	[51]
Difference-in-differences	As long as the naturally occurring changes over time in the intervention and control group are the same, the difference in the change in the outcome between both groups can be interpreted as the intervention effect.	Repeated cross-sectional	Observed and time-invariant unobserved confounders	Vulnerable to violation of the common trend assumption	[22]
Fixed effects	Multiple observations within units are compared, such as repeated measurements over time within individuals. Effects of unobserved confounders that differ between units but remain constant over time are eliminated.	Longitudinal	Observed and time-invariant unobserved confounders	Vulnerable to unobserved time-variant confounders; Knocks out all cross-sectional variations between units; Susceptible to measurement errors over time;	[52, 53]
Instrumental variable approach	An instrument creates variation in exposure to the intervention, without being directly related to the outcome itself.	Cross-sectional	Observed and unobserved confounders	Difficult to find good instrumental variables; Exogeneity of instruments cannot be easily tested; Weak instruments and finite samples might result in bias; Local average treatment effect problem;	[54]
Regression discontinuity	As long as the association between a variable and an outcome is smooth, any discontinuity in the outcome after a cut-off point of this variable can be regarded as an intervention effect.	Cross-sectional	Observed and unobserved confounders	Low external validity; Local average treatment effect problem in a fuzzy design;	[23]
Interrupted time-series	Identification of a sudden change in level of the health outcome (a change of intercept) or a more sustained change in trend of the health outcome (a change of slope) around the time of the implementation of the intervention.	Repeated measures	Observed confounders	Difficult to evaluate the interventions implemented slowly, or need unpredictable time to be effective; Vulnerable to other external interventions or shocks within the period;	[55–57]

1. *Regression adjustment:* Standard multivariate regression techniques allow investigating the effect of a policy by adjusting the association between policy exposure and health outcomes for observed differences between those exposed and unexposed to the policy in the prevalence of confounding factors. Theoretically, if all possible confounders can be controlled for, the estimated policy effect will be unbiased. It is unrealistic to assume, however, that all possible confounders can be measured.
  We illustrate this method using data obtained after the policy only (Health_t2_), because this method is often applied in situations where data obtained before the policy are not available.
2. *Propensity score matching:* Propensity score matching involves estimating the ‘propensity’ or likelihood that each person or group has of being exposed to the policy, based on a number of known characteristics, and then matching exposed to unexposed individuals based on similar levels of the propensity score. Propensity score matching assumes that for a given propensity score, exposure to the policy is random. It is similar to regression analysis with control for confounding in that it aims to reduce bias due to observed confounding variables. It is different from regression adjustment, because matching yields a parameter for the average impact over the subspace of the distribution of all covariates that are both represented among the treated and the control groups (i.e. only for the space where there is “common support”).
  We illustrate this method also with data obtained after the policy (Health_t2_), because this method is often applied in situations where data before the policy are not available.
3. *Difference-in-differences analysis:* Difference-in-differences analysis compares the change in outcome for an exposed group between a moment before and a moment after the implementation of a policy to the change in outcome over the same time period for a non-exposed group. The two groups may have different levels of the outcome before the policy, but as long as any ‘naturally occurring’ changes over time can be expected to be the same for both, the difference in the change in outcome between the exposed and non-exposed groups will be an unbiased estimate of the policy effect.
  In order to illustrate this technique, we had to slightly modify our data example. Thus far, we only used data after the implementation of the policy. For the difference-in-differences analysis, we assumed that the data in our example had been collected in a repeated cross-sectional design.
4. *Fixed effects analysis*: Fixed effects analysis compares multiple observations within the same individuals or groups over time, and reveals the average change in the outcome due to the policy. Because each individual or group is compared with itself over time, differences between individuals or groups that remain constant over time – even if unmeasured – are eliminated and cannot confound the results. Numerically, fixed effects analysis produces the same results as adding a dummy variable for each individual or group into standard multivariate regressions.
  In order to illustrate the fixed effects analysis, we considered our fictitious dataset to be a longitudinal dataset with repeated measures of self-assessed health before and after the implementation of the policy.
5. *Instrumental variable analysis:* Instrumental variable analysis involves identifying a variable predictive of exposure to the policy, which in itself has no direct relationship with the outcome except through its effects on policy exposure or through other variables which have been adjusted in the regression. The technique uses the variation in outcome generated by this ‘instrument’ to test whether exposure to the policy is related to the outcome.
  We illustrate the instrumental variable approach with the cross-sectional data obtained after the policy. We constructed an instrument which is predictive of exposure to the policy and not directly related to health.
6. *Regression discontinuity:* Regression discontinuity is a form of analysis that can be used when areas or individuals are assigned to a policy depending on a cut-off point of a continuous measure. The basic idea is that, conditional on the relationship between the assignment variable and the outcome, the exposure to the policy at the cut-off point is as good as random, comparing health outcomes of those just below and just above the cut-off point provides an estimate of the effect of the policy.
  To illustrate the application of regression discontinuity, we created a new dataset. The main reason was the need to create a “threshold”, and thereby to introduce a new variable, distinguishing persons who could receive the policy from those who were not eligible for it.
7. *Interrupted time-series:* Where time-series data are available and there is a clear-cut change in policy at a specific point in time, interrupted time-series analysis can be used to estimate the policy effect. Regression analysis is used to detect any sudden change in level of the health outcome (in regression terms: a change of intercept) or a more sustained change in the trend of the health outcome (in regression terms: a change of slope) around the time the policy is implemented. The analysis estimates the policy effect by comparing the health outcomes before and after policy implementation. Interrupted time series is different from a difference-in-differences analysis, because interrupted time series analysis does not need a separate control group. In fact, it uses its own trend before the implementation of the policy as the control group.
  To illustrate this method, we generated a time-series dataset which contained 40 years of observations. The quantitative characteristics of the dataset are similar to those used in the other calculation examples.

### Statistical assessment of the impact of NPE in terms of socioeconomic inequalities in health

Analytically, assessing to what extent a policy does have an effect in lower and higher socioeconomic groups can be done in two ways. The first is to conduct a stratified analysis, using socioeconomic position as a stratification variable, resulting in policy effects for both lower and higher socioeconomic groups. The second is to include an interaction term between the variable for policy exposure and the indicator of socioeconomic position. For the latter, if the confounding effects of other covariates differ between socioeconomic groups, interaction terms between the indicator of socioeconomic position and these covariates also need to be added. If all interactions are included, the policy effects derived from an analysis stratified by socioeconomic position and from an analysis with interaction terms will be the same. For illustrative purpose, we included all the interactions in our analysis so that the results from interaction terms and stratified analysis were the same.

Most of the techniques described above require a regression analysis. Whereas a linear regression analysis results in an absolute effect of the policy, a logistic regression analysis results in a relative policy effect. Propensity score matching uses a pair-matched difference in the outcome to quantify the policy effect.

For those techniques resulting in a policy effect among the exposed only (all techniques described above, except interrupted time series), we then need to combine these effects with the proportion of exposed persons in higher and lower socioeconomic groups, in order to calculate the impact of policy on absolute and relative inequalities among the whole population. Currently, there is no prescribed statistical procedure to do this. Our approach is to calculate the prevalence of people having poor health in each educational group after the policy (an observed prevalence) and the predicted prevalence of people having poor health in absence of the policy (a predicted prevalence). The latter can be calculated by excluding the coefficient for the policy assignment from the equation, while keeping all other coefficients in the model the same. With the observed and predicted prevalence rates, absolute rate differences and relative rate ratios can be calculated. The differences in the absolute rate differences or the relative rate ratios with and without the policy then show the impact of the policy on the magnitude of health inequalities. Bootstrapping with 1000 replications was used to calculate the confidence intervals around the estimated impact of a policy. All analyses were performed in Stata 13.1.

## Results

### Regression adjustment

In a stratified analysis, the effect of the policy can be modeled for those in higher and lower educational groups separately, adjusting for gender as a confounder:

$$ \mathrm{Healt}{\mathrm{h}}_{\mathrm{i}\mathrm{t}2} = {\upbeta}_0 + {\upbeta}_1\mathrm{polic}{\mathrm{y}}_{\mathrm{i}} + {\upbeta}_2\mathrm{gende}{\mathrm{r}}_{\mathrm{i}} + {\upmu}_{\mathrm{i}} $$

where health_it2_ is the health of individual i in year t2, β_0_ is the intercept, β_1_ and β_2_ are regression coefficients and μ_i_ is the error term.

If we use logistic regression, which is appropriate in situations with a binary health outcome as in our example, the odds ratio for the policy effect can be calculated from β_1_ and represents the higher or lower odds of having poor health after the policy for those exposed to the policy as compared to those unexposed to the policy. Because gender in this example is the only confounder, and because we were able to measure and adjust for it, the odds ratio can be interpreted as the policy effect. Table 3 shows these policy effects for people in lower and higher educational groups. The policy effect is essentially similar for people in lower (OR = 0.647, 95% CI [0.570, 0.734]) and higher educational groups (OR = 0.679, 95% CI [0.550, 0.839]). Please note that this analysis gives us estimates of relative rather than absolute policy effects. The discrepancy between the estimated odds ratios for the policy effect (0.647 and 0.679) on the one hand and the policy effects that we imposed in the dataset (a reduction of the probability of poor health among the exposed as compared to the unexposed of 30% for people in both higher and lower educational groups) on the other hand is due to the logistic transformation.

**Table 3:** Policy effects derived from the seven methods based on education-stratified analysis and the inclusion of interaction terms

Method	Specification	Low-educated [95% CI]	High-educated [95% CI]	Interaction term [95% CI]
Regression adjustment	Logistic regression, adjusted for gender	0.647 [0.570, 0.734] (odds ratio)	0.679 [0.550, 0.839] (odds ratio)	0.953 [0.745, 1.218] (odds ratio)
Propensity score matching	Matched on gender	−0.048 [-0.065, -0.031] (probability difference)	−0.020 [-0.031, -0.009] (probability difference)	Not applicable
Difference-in-differences	Logistic regression	0.666 [0.574, 0.773] (odds ratio)	0.687 [0.530, 0.890] (odds ratio)	0.970 [0.719, 1.307] (odds ratio)
Fixed effects	Linear regression, adjusted for time	−0.044 [-0.051, -0.037] (probability difference)	−0.016 [-0.023, -0.009] (probability difference)	−0.029 [-0.039, -0.019] (probability difference)
Instrumental variable	Probit regression	−0.050 [-0.063, -0.037] (probability difference)	−0.020 [-0.029, -0.011] (probability difference)	−0.036 [-0.057, -0.015] (probability difference)
Regression discontinuity	Logistic regression around the income threshold	0.678 [0.495, 0.929] (odds ratio)	0.687 [0.483, 0.977] (odds ratio)	0.987 [0.615, 1.583] (odds ratio)
Interrupted time-series	Linear regression	−0.023 [-0.027,-0.020] (probability difference)	−0.005 [-0.008, -0.002] (probability difference)	−0.019 [-0.023, -0.014] (probability difference)

Regression analysis also allows us to introduce an interaction term between (low) education and exposure to the policy (“low-edu*policy”):

$$ \mathrm{Healt}{\mathrm{h}}_{\mathrm{i}\mathrm{t}2} = {\upbeta}_0 + {\upbeta}_1\mathrm{polic}{\mathrm{y}}_{\mathrm{i}} + {\upbeta}_2\mathrm{gende}{\mathrm{r}}_{\mathrm{i}} + {\upbeta}_3\mathrm{low}\hbox{-} \mathrm{e}\mathrm{d}{\mathrm{u}}_{\mathrm{i}} + {\upbeta}_4\left(\mathrm{low}\hbox{-} \mathrm{ed}{\mathrm{u}}_{\mathrm{i}}*\mathrm{polic}{\mathrm{y}}_{\mathrm{i}}\right) + {\upbeta}_5\left(\mathrm{low}\hbox{-} \mathrm{ed}{\mathrm{u}}_{\mathrm{i}}*\mathrm{gende}{\mathrm{r}}_{\mathrm{i}}\right) + {\upmu}_{\mathrm{i}} $$

where β_0_ is the intercept, β_1,_ β_2,_ β_3,_ β_4_ and β_5_ are regression coefficients and μ_i_ is the error term.

As shown in Table 3, the interaction term between the policy and education (β_4_) was not statistically significant (0.953, 95% CI [0.745, 1.218]). This indicates that we cannot show that the policy effects for people in lower and higher educational groups are different, which is in line with the findings from the stratified analysis.

These results only represent the relative policy effect for people exposed as compared to those unexposed to the policy; they do not take into account the proportion of exposed people in each educational group. To do so, we had to apply an extra step. Using the stratified analyses, we calculated the predicted prevalence of poor health if the policy would have not been implemented (please note that we could have also used the analysis with the interaction terms; if all interactions are included this will provide exactly the same results). This was done by leaving out the term for the policy, keeping all other coefficients in the regression equations, and computing the predicted prevalence of poor health. Subsequently, we calculated the rate difference between people in higher and lower educational groups using the observed prevalence (for the situation in which the policy was implemented), and the predicted prevalence (for the situation in which the policy would not have been implemented) (Table 4). For example, the rate difference in the situation with the policy effect was 9.14% (16.63–7.49) and was 11.11% without the policy. In a similar way, the rate ratios were also calculated for both situations. The impact of the policy on health inequality could now be measured (1) as the change in absolute inequality (e.g., as the change in the rate difference) or (2) as a change in relative inequality (e.g., as a change in the rate ratio). In our example, the change in the rate difference is 1.97% points (11.11–9.14%) which means that the policy reduced the absolute inequality between people in lower and higher educational groups by almost 2% points (Table 5). Further, the change in the rate ratio was 12.2% ((2.39–2.22)/(2.39–1)). This means that the policy reduced the relative inequality by more than 12%. We have also calculated the confidence intervals of these estimates (Table 5).

**Table 4:** Observed and predicted prevalence of poor health, rate difference and rate ratio for low and high educated groups with and without the implementation of the policy, as obtained using the seven methods

	Low-educated (%)	High-educated (%)	Rate difference	Rate ratio
Observed prevalence with policy effect	16.63	7.49	9.14	2.22
Predicted prevalence without the policy effect^a^				
Regression adjustment	19.11	8.00	11.11	2.39
Propensity score matching	19.03	7.99	11.04	2.38
Difference-in-differences analysis	18.97	7.98	10.99	2.38
Fixed effects models	18.84	7.88	10.96	2.39
Instrumental variable analysis	19.15	7.99	11.16	2.40
Regression discontinuity	Not comparable	Not comparable	Not comparable	Not comparable
Interrupted time-series	18.96	7.97	10.99	2.38

^a^As derived from the stratified analyses, reported as proportion of individuals with poor health (or, equivalently, individual probability of having poor health)

**Table 5:** Summary table of the policy effect on absolute and relative inequalities in health

Method	Estimated policy effect on absolute health inequality^a^(reduced rate difference in % points, [95% CI])	Estimated policy effect on relative health inequality^b^ (reduced rate ratio, in %, [95% CI])
1. Regression adjustment	1.97 [1.19, 2.76]	12.20 [4.49, 19.90]
2. Matching	1.89 [1.77, 2.02]	11.60 [8.99, 14.20]
3. Difference-in-differences	1.85 [0.88, 2.82]	11.33 [1.37, 21.29]
4. Fixed effects	1.82 [1.28, 2.36]	12.26 [5.45, 19.08]
5. Instrumental variable	2.02 [1.34, 2.69]	12.62 [6.07, 19.17]
6. Regression discontinuity	not comparable	not comparable
7. Interrupted time-series	1.85 [1.45, 2.26]	11.53 [6.05, 17.00]
Real policy effect	1.86	11.25
Simple before-and-after comparison	0.86	−22.03

^a^We calculated the prevalence of people having poor health in each educational group following the real policy implementation and the predicted prevalence if leaving out the term for the policy effect (when there was no policy). The reported numbers represent the absolute reduction of the rate difference that can be attributed to the policy

^b^The reported numbers represent the relative reduction of the rate ratio (RR) calculated as follows: (RR_without policy_ – RR_with policy_)/(RR_without policy_ ‐ 1) * 100

### Propensity score matching

In order to obtain an estimate for the effect of the policy on health inequalities we conducted a stratified analysis, i.e. we applied propensity score matching within the high and low educational groups separately. The first step in the analysis was to calculate the “propensity” of being exposed to the policy. Logistic regression analysis, with being exposed or not as the binary outcome and gender as the predictor, was used to calculate the propensity of being exposed. Individuals with the same propensity who were indeed exposed to the policy could then be matched with individuals with almost the same (“the nearest neighbor”) propensity who were not exposed to the policy.

The policy effect was estimated as the average of the differences in the probability of poor health within matched pairs of exposed and unexposed individuals. This produces an absolute measure of the policy effect. Table 3 lists the results obtained from the propensity score matching analysis for people in lower and higher educational groups separately. For people in lower and higher educational groups, the policy reduced the probability of having poor health among exposed individuals by almost 5 percentage points (-0.048) and 2 percentage points (-0.020), respectively. Although we imposed the same relative policy effect regardless of the education level in the data, the absolute effect of the policy was larger for people in lower educational group than for people in higher educational group, because the prevalence of poor health before the policy was higher among the lower educational group.

To calculate the absolute decrease of the prevalence of poor health, the effect of the policy for people in lower and higher educational groups should be multiplied with the proportion of persons exposed to the policy in each educational group. Among all people in lower educational group, regardless of whether they were exposed or not to the policy, the probability of having poor health declined by 2.5 percentage points ((-0.048)*(5000/10,000) = -0.024). Among all the people in higher educational group, the probability of having poor health declined by 0.5 percentage points ((-0.020)*(2500/10,000) = -0.005).

In order to estimate the effect of the policy on the magnitude of health inequalities, we need the rate difference and rate ratio in a scenario with and in a scenario without the policy effect. In a scenario without the policy effect, the predicted prevalence of having poor health for people in lower educational group is the observed prevalence (16.63%) plus the reduction as a result of the policy (2.4%), which is then 19.03%. For people in higher educational group, the prevalence is 7.99% (7.49% +0.5%).

The rate difference in the scenario with the policy was 9.14% (16.63–7.49); in the scenario without the policy it was 11.04% (19.03–7.99). This means that the policy reduced the absolute inequality in poor health by almost 2%. The rate ratio in the scenario with the policy was 2.22 (16.63/7.49); in the scenario without the policy it was 2.38 (19.03/7.99). This means that the policy reduced the relative inequality of poor health by almost 12%.

In propensity score matching, the policy effect is indicated by the average difference between the exposed and the matched unexposed individuals. There is no regression equation in the matching process, and therefore it was considered impossible to use an interaction term in a propensity matching analysis.

### Difference-in-differences analysis

In the analysis, we modeled health (measured both before and after implementation of the policy) as a function of exposure to the policy, time, and an interaction between exposure to the policy and time. By allowing levels of health to be different between exposed and unexposed before the policy, the technique accounts for unobservable confounding by time-invariant characteristics that differ in their prevalence between the exposed and unexposed. In our example ‘gender’ was not controlled for, and therefore acted as an unobservable confounder.

In a stratified analysis, the model to be used for people in lower and higher educational groups separately is:

$$ \mathrm{Healt}{\mathrm{h}}_{\mathrm{i}\mathrm{t}} = {\upbeta}_0 + {\upbeta}_1\mathrm{polic}{\mathrm{y}}_{\mathrm{i}} + {\upbeta}_2\mathrm{y}\mathrm{e}\mathrm{a}{\mathrm{r}}_{\mathrm{t}} + {\upbeta}_3\left(\mathrm{polic}{\mathrm{y}}_{\mathrm{i}}*\mathrm{yea}{\mathrm{r}}_{\mathrm{t}}\right) + {\upmu}_{\mathrm{i}\mathrm{t}} $$

where health_it_ is the health of individual i in year t, β_0_ is the intercept, β_1_, β_2_ and β_3_ are regression coefficients and μ_it_ is the error term.

If we again use logistic regression, the coefficient for the variable “policy” (β_1_) now measures the relatively higher or lower odds of having poor health for those exposed as compared to those unexposed to the policy before the implementation of the policy (which in our example was driven by the fact that women were in better health before the implementation and more exposed to the policy). The coefficient for the variable “year” (β_2_) represents the naturally occurring change in health over time among the unexposed. The coefficient for the interaction term “policy*year” (β_3_) indicates the policy effect, i.e. the difference in change of poor health over time between the unexposed and exposed. Table 3 shows the policy effects for people in lower and higher educational groups. The relative policy effect is essentially similar for people in lower educational group (OR = 0.666, 95% CI [0.574; 0.773]) and for people in higher educational group (OR = 0.687, 95% CI [0.530; 0.890]). This is again in line with what we imposed in the data.

In a difference-in-difference analysis, we can also introduce a three-way interaction term between policy, year and low education:

$$ \begin{array}{l}\mathrm{Healt}{\mathrm{h}}_{\mathrm{i}\mathrm{t}} = {\upbeta}_0 + {\upbeta}_1\mathrm{polic}{\mathrm{y}}_{\mathrm{i}} + {\upbeta}_2\mathrm{yea}{\mathrm{r}}_{\mathrm{t}} + {\upbeta}_3\left(\mathrm{polic}{\mathrm{y}}_{\mathrm{i}}*\mathrm{yea}{\mathrm{r}}_{\mathrm{t}}\right) + {\upbeta}_4\left(\mathrm{low}\hbox{-} \mathrm{ed}{\mathrm{u}}_{\mathrm{i}}\right)\ \\ {} + {\upbeta}_5\left(\mathrm{low}\hbox{-} \mathrm{ed}{\mathrm{u}}_{\mathrm{i}}*\mathrm{polic}{\mathrm{y}}_{\mathrm{i}}\right) + {\upbeta}_6\left(\mathrm{low}\hbox{-} \mathrm{ed}{\mathrm{u}}_{\mathrm{i}}*\mathrm{yea}{\mathrm{r}}_{\mathrm{t}}\right) + {\upbeta}_7\left(\mathrm{low}\hbox{-} \mathrm{ed}{\mathrm{u}}_{\mathrm{i}}*\mathrm{polic}{\mathrm{y}}_{\mathrm{i}}*\mathrm{yea}{\mathrm{r}}_{\mathrm{t}}\right) + {\upmu}_{\mathrm{i}\mathrm{t}}\end{array} $$

where health_it_ is the health of individual i in year t, β_0_ is the intercept, β_1_ - β_7_ are regression coefficients and μ_it_ is the error term.

The three-way interaction labeled “*low-edu*_*i*_ *policy_*i*_ *year_*t*_” (β_7_) indicates the differential policy effect for people in lower and higher educational groups. As shown in Table 3, this interaction term was not statistically significant (OR = 0.970, 95% CI [0.719; 1.307]). Thus, the policy effect was not significantly different for people in lower and higher educational groups, which corresponds to what we have imposed in the data example.

Using a similar approach as for the regression adjustment, and again based on the stratified analyses, we subsequently calculated the predicted prevalence of poor health if the policy would not have been implemented. It allowed us to calculate the rate differences between people in lower and higher educational groups based on the predicted prevalence (if the policy would not have been implemented) as well as the rate ratios. As shown in Table 4, the policy effect on absolute health inequalities (e.g. the change in the rate differences) was 1.85% (10.99-9.14). This means that the policy reduced the rate difference between people in lower and higher educational groups by almost 2 percentage points. Similarly, we can calculate the policy effect on relative health inequality as the change in the rate ratio, resulting in the finding that the policy reduced the relative excess risk of the people in lower educational group by more than 11%.

### Fixed effects model

With a binary outcome, one could use logistic regression analysis. However, in fixed effects logistic regression analysis, observations with the same health status in two (or more) periods will be excluded from the analysis; only the within-unit variations over time will be used. Therefore, a large part of the observations in our dataset would be excluded. While logistic regression analysis would produce valid estimates, it would lead to results that cannot be compared to those obtained from the other methods. For reasons of comparability, we used linear probability regressions with fixed effects, which also produces valid estimates. 1Again, we treated ‘gender’ as an unobserved confounder.

Linear probability regression was used, in which the coefficient for the policy (β_1_ in the formula below) represented an absolute change in the probability of having poor health as a result of exposure to the policy. In a stratified analysis, this can be modeled as follows for those in higher and lower educational groups separately:

$$ \mathrm{Healt}{\mathrm{h}}_{\mathrm{i}\mathrm{t}} = {\upbeta}_0 + {\upbeta}_1\mathrm{polic}{\mathrm{y}}_{\mathrm{i}\mathrm{t}} + {\upbeta}_2\mathrm{y}\mathrm{e}\mathrm{a}{\mathrm{r}}_{\mathrm{t}} + {\mathrm{d}}_{\mathrm{i}} + {\upmu}_{\mathrm{i}\mathrm{t}} $$

where health_it_ is health of individual i in year t, β_0_ is the intercept_,_ β_1_ and β_2_ are regression coefficients, d_i_ is a set of individual dummy variables and μ_it_ is the error term.

Table 3 shows that the absolute policy effect is larger among people in lower educational group (*β*_*1*=_-0.044, 95% CI [-0.051; -0.037]) than among people in higher educational group (*β*_*1=*_-0.016, 95% CI [-0.023; -0.009]).

Fixed effects analysis also allows us to introduce an interaction term between (low) education and exposure to the policy:

$$ \mathrm{Healt}{\mathrm{h}}_{\mathrm{i}\mathrm{t}} = {\upbeta}_0 + {\upbeta}_1\mathrm{polic}{\mathrm{y}}_{\mathrm{i}\mathrm{t}} + {\upbeta}_2\mathrm{y}\mathrm{e}\mathrm{a}{\mathrm{r}}_{\mathrm{t}} + {\upbeta}_3\left(\mathrm{low}\hbox{-} \mathrm{ed}{\mathrm{u}}_{\mathrm{i}\mathrm{t}}*\mathrm{polic}{\mathrm{y}}_{\mathrm{i}\mathrm{t}}\right)+{\upbeta}_4\left(\mathrm{low}\hbox{-} \mathrm{ed}{\mathrm{u}}_{\mathrm{i}\mathrm{t}}*\mathrm{yea}{\mathrm{r}}_{\mathrm{t}}\right) + {\mathrm{d}}_{\mathrm{i}} + {\upmu}_{\mathrm{i}\mathrm{t}} $$

where health_it_ is health of individual i in year t, β_0_ is the intercept_,_ β_1_ – β_4_ are regression coefficients, d_i_ is a set of individual dummy variables and μ_it_ is the error term.

As shown in Table 3, the interaction term for low education and policy (“low-edu*policy”) is statistically significant (*β*_*3*_ = -0.029, 95% CI [-0.039; -0.019]), which indicates that the policy effect is indeed different between people in lower and higher educational groups. The negative sign of the coefficient for the interaction term indicates that the absolute policy effect is larger among people in lower educational group, as was also found in the stratified analysis.

Again we can use the fitted values to estimate the policy effect on health inequalities. Based on the results in Table 4, we can calculate the policy effect on absolute health inequality as the change in the rate differences: 10.96–9.14 = 1.82. This means that the policy has reduced the rate difference between people in lower and higher educational groups by almost 2 percentage points. Similarly, we can calculate the policy effect on relative health inequality, which then results in the finding that the policy reduced the relative excess risk of people in lower as compared to higher educational group by more than 12%.

### Instrumental variable analysis

Again, we used gender as an unobserved confounder. In a straightforward regression analysis, exposure to the policy would be endogenous (as gender would determine exposure to the policy to some extent, and is now included in the error term), and as a consequence the estimated effect of policy on health would be biased. We therefore used an instrument, e.g. the “distance from the house of the respondent to the closest free medical service”. For this to be a valid instrument, it should be clearly predictive of exposure to the policy, related to health via the policy (use of the free medical service) only, and not be related to any unmeasured confounder (information about the construction of the instrumental variable used in our analyses is available upon request).

The instrumental variable analysis was conducted in a two-stage least squares regression. The basic idea of this analysis in our example was to first regress the policy exposure on the instrumental variable in order to capture the variation in policy exposure induced by the instrument, and to subsequently regress the health outcome on the predicted values for policy exposure. The instrumental variable analysis with logistic regression cannot be easily conducted in Stata, and therefore we used probit regression (specifically “ivprobit”). The coefficients from the probit regressions were transformed into marginal effects to make them comparable to those from linear regressions.

While the approach is intuitively easy if stratified by education, it is more complicated for an analysis using the interaction between policy and education. Because exposure to the policy is endogenous, the interaction between education and policy exposure is endogenous as well. This requires an instrument for the interaction terms as well; we used the interaction between education and distance from home to the closest free medical service for this purpose. In the first step of the two stage regression, both instruments predict exposure to the policy as well as the interaction between education and exposure to the policy. The predicted values are then used in the second stage of regression, resulting in unbiased effects of exposure to the policy and the interaction between policy exposure and education on health.

Table 3 shows that the absolute policy effect is larger among people in lower educational group (β = -0.050, 95% CI [-0.063; -0.037]) than among people in higher educational group (β = -0.020, 95% CI [-0.029; -0.011]). The interaction term for low education and policy was statistically significant (β = -0.036; 95% CI [-0.057; -0.015]), which indicated that the policy effect was different between people in lower and higher educational groups indeed.

As for the other methods, we used the predicted values from the regression analysis (in this case, the second stage of the analysis) to estimate the policy effect on health inequality (Table 4). Using the values in Table 4, we calculated the policy effect on absolute health inequalities as the change of the rate difference: 11.16–9.14 = 2.02%. This means that the policy reduced the rate difference between people in lower and higher educational groups by 2 percentage points. Similarly, we calculated the policy effect on relative health inequalities as the change of the rate ratio and found that the policy reduced the relative excess risk of poor health among people in lower educational group by almost 13%.

### Regression discontinuity

Here we had to create a new dataset with a “threshold” distinguishing persons who could receive the policy from those who were not eligible for it. For this purpose, we created a sharp regression discontinuity design with income as the “threshold” variable: those with a household income of less than 2000 euros per month could receive the free medical care, whereas those with higher incomes were not eligible to receive the free medical care. We assumed that the sharp threshold of 2000 euro resulted in a ‘sharp’ regression discontinuity, without changing the effect of income on health. Because people in lower educational group generally tend to have lower household incomes, more people in lower educational group were exposed to the policy. The imposed policy effect was still a reduction of the prevalence of poor health by 30% regardless of education level. The dataset created contained cross-sectional data after the implementation of the policy. Details about the generation of the data for the regression discontinuity are available upon request.

In a stratified analysis, this was modeled as follows for individuals in higher and lower educational groups separately:

$$ \mathrm{Healt}{\mathrm{h}}_{\mathrm{i}} = {\upbeta}_0 + {\upbeta}_1\left(\mathrm{incom}{\mathrm{e}}_{\mathrm{i}}\hbox{-} 2000\right) + {\upbeta}_2\mathrm{polic}{\mathrm{y}}_{\mathrm{i}} + {\upmu}_{\mathrm{i}} $$

where health_i_ is health of individual i, β_0_ is the intercept, β_1_ and β_2_ are regression coefficients, and μ_i_ is the error term.

Individual-level health was still the health outcome. The value for the variable “policy” was 1 if the individual’s monthly income was equal to or less than 2000 euro per month. The regression coefficient β_1_ reflects the average effect of income on health. The regression coefficient β_2_ reflects the discontinuity in health which was caused by the implementation of the policy. The analysis was done among individuals whose monthly income is around 2000 (e.g. individuals whose monthly income is between 1800 and 2200). Using logistic regression, the odds ratio resulting from the coefficient for the variable “policy” (β_2_) measured the higher or lower odds of having poor health between those exposed to the policy and those not exposed to the policy. Table 3 shows that the relative policy effect is similar for people in lower educational group (OR = 0.678, 95% CI [0.495; 0.929]) and for people in higher educational group (OR = 0.687, 95% CI [0.483; 0.977]). Approximately, this is the 30% chance of reversing from poor to good health regardless of education level, as imposed in the data.

Regression discontinuity analysis also allows us to introduce interaction terms:

$$ \begin{array}{l}\mathrm{Healt}{\mathrm{h}}_{\mathrm{i}} = {\upbeta}_0 + {\upbeta}_1\left(\mathrm{incom}{\mathrm{e}}_{\mathrm{i}}\hbox{-} 2000\right) + {\upbeta}_2\mathrm{polic}{\mathrm{y}}_{\mathrm{i}} + {\upbeta}_3\mathrm{low}\hbox{-} \mathrm{ed}{\mathrm{u}}_{\mathrm{i}} + {\upbeta}_4\left(\mathrm{low}\hbox{-} \mathrm{ed}{\mathrm{u}}_{\mathrm{i}}*\left(\mathrm{incom}{\mathrm{e}}_{\mathrm{i}}\hbox{-} 2000\right)\right)\ \\ {} + {\upbeta}_5\left(\mathrm{low}\hbox{-} \mathrm{ed}{\mathrm{u}}_{\mathrm{i}}*\mathrm{polic}{\mathrm{y}}_{\mathrm{i}}\right) + {\upmu}_{\mathrm{i}}\end{array} $$

where health_i_ is health of individual i, β_0_ is the intercept, β_1_ – β_5_ are regression coefficients, and μ_i_ is the error term.

As shown in Table 3, the interaction term for low education and policy (“low-edu*policy”) is statistically insignificant (OR = 0.987, 95% CI [0.615; 1.583]), which indicates that the policy effect is not statistically different between people in lower and higher educational groups.

**Fig. 1:**
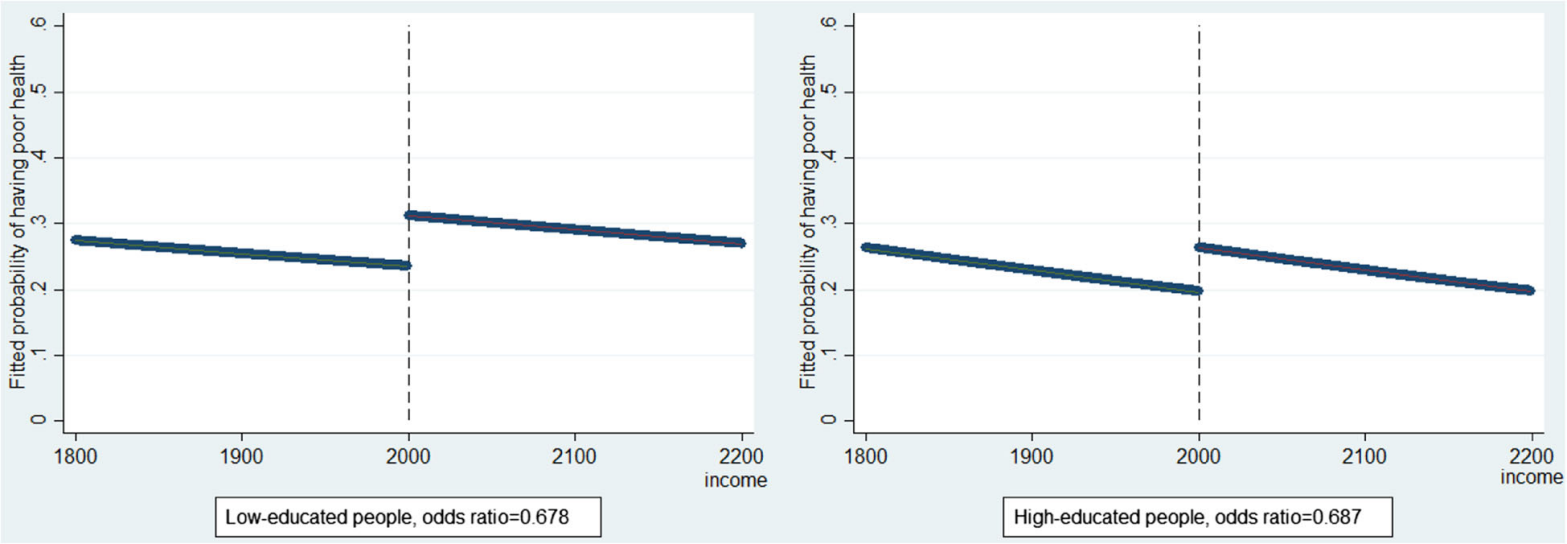
Results from regression discontinuity by education

Results from the regression discontinuity analysis are also reported in a graphical way (Fig. 1). In Fig. 1, similar discontinuities around the income level of 2000 euro per month were observed among people in lower and higher educational groups. This indicates similar instant policy effects. In our example, although the policy effects were independent of educational level, more people in lower educational group were exposed to the policy, leading to a decreased health inequality. However, this cannot be shown in the figure.

Again we can use the fitted values to estimate the policy effect on health inequalities and follow the same steps to calculate the changes in absolute and relative inequalities as a result of the policy. However, as the analysis was only performed based on the observations around the cutoff point of 2000 euro per month (e.g. 1800–2200 euro per month), we could not produce the policy effect on health inequalities among the whole population. This is a characteristic of the regression discontinuity method, and should not be seen as a failure of the example. Given that we generated a different setting for this method and the estimated policy effects only represented the policy effects among a part of the whole population, the calculated policy effects on health inequalities were not comparable to those from other methods and we therefore did not present them in Tables 4 and 5.

### Interrupted time series

Here we generated a time-series dataset which contained 40 years of observations. Because this method (in our example) uses aggregate data, we could consider our health outcome, the prevalence of poor health, to be continuous (as opposed to binary in the other examples). For people in lower educational group, the prevalence of poor health decreased by around 0.1 percentage points each year before the policy. For people in higher educational group, the prevalence of poor health decreased by around 0.2 percentage points each year before the policy. The policy was implemented half way during the period of observation (i.e. year 20). For reasons of simplicity, we assumed that the policy affected the level of health (the intercept) immediately after its implementation. Details about the way of generating the data are available upon request.

In a stratified analysis, the model used for individuals in higher and lower educational groups separately was:

$$ \mathrm{Healt}{\mathrm{h}}_{\mathrm{t}} = {\upbeta}_0 + {\upbeta}_1\mathrm{y}\mathrm{e}\mathrm{a}{\mathrm{r}}_{\mathrm{t}} + {\upbeta}_2\mathrm{polic}{\mathrm{y}}_{\mathrm{t}} + {\upbeta}_3{\left(\mathrm{year}\ \mathrm{a}\mathrm{fter}\ \mathrm{policy}\right)}_{\mathrm{t}} + {\upmu}_{\mathrm{t}} $$

where health is the prevalence of self-assessed health, β_0_ is the intercept_,_ β_1_ and β_2_ are regression coefficients, and μ_t_ is the error term.

The variable “year” represented the calendar years and ranged from 1 to 40. The variable “policy” was a dummy variable with value 1 if it was larger than 20, and value 0 if it is smaller or equal to 20. The variable “year after policy” was the number of years after the implementation of the policy. In the regression, the coefficient of “year” (β_1_) indicated the natural trend before the policy. The coefficients for “policy” and “year after policy” represented the change in the intercept and the change in the slope due to the policy.

**Fig. 2:**
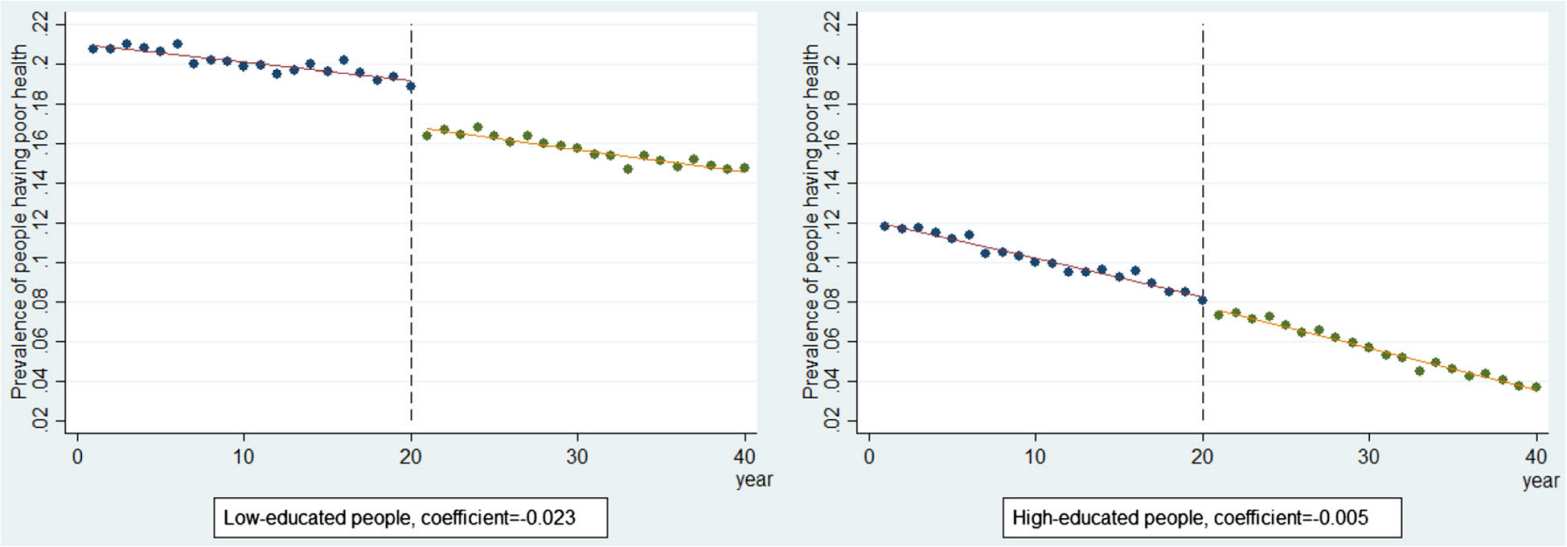
Results from interrupted time-series by education

Figure 2 presents the results of the interrupted time-series analysis, stratified by education. As mentioned above, aggregated data were used, which already incorporated the effect of both the real policy effect on the exposed people and the proportion of exposed people in lower and higher educational groups. Since more l people in lower educational group were exposed, an instant effect on reducing health inequalities was observed, indicated by a larger drop in the prevalence of poor health in the lower educational group directly after the implementation of the intervention in year 20. As shown in table 3, the policy reduced the prevalence of poor health for people in lower educational group immediately by 2.3% points and it reduced the prevalence of poor health for people in higher educational group immediately by 0.5% points.

Interrupted time-series analysis also allows us to introduce interaction terms:

$$ \begin{array}{l}\mathrm{Healt}{\mathrm{h}}_{\mathrm{t}} = {\upbeta}_0 + {\upbeta}_1\mathrm{yea}{\mathrm{r}}_{\mathrm{t}} + {\upbeta}_2\mathrm{polic}{\mathrm{y}}_{\mathrm{t}} + {\upbeta}_3{\left(\mathrm{year}\ \mathrm{after}\ \mathrm{policy}\right)}_{\mathrm{t}} + {\upbeta}_4\mathrm{low}\hbox{-} \mathrm{ed}{\mathrm{u}}_{\mathrm{t}} + {\upbeta}_5\left(\mathrm{low}\hbox{-} \mathrm{ed}{\mathrm{u}}_{\mathrm{t}}*\mathrm{yea}{\mathrm{r}}_{\mathrm{t}}\right)\ \\ {} + {\upbeta}_6\left(\mathrm{low}\hbox{-} \mathrm{ed}{\mathrm{u}}_{\mathrm{t}}*\mathrm{polic}{\mathrm{y}}_{\mathrm{t}}\right) + {\upbeta}_7\left(\mathrm{low}\hbox{-} \mathrm{ed}{\mathrm{u}}_{\mathrm{t}}*{\left(\mathrm{year}\ \mathrm{after}\ \mathrm{policy}\right)}_{\mathrm{t}}\right) + {\upmu}_{\mathrm{t}}\end{array} $$

where health is the prevalence of self-assessed health, β_0_ is the intercept_,_ β_1_ – β_7_ are regression coefficients, and μ_t_ is the error term.

The coefficients for “low-edu*policy” represent the change in the intercept due to the policy. As shown in Table 3, the interaction “low-edu*policy” is statistically significant (coefficient = -0.019), which suggests that the policy effect is larger among lower educational group.

As before, using the values in Table 4 we calculated the policy effect on absolute health inequality as the change of the rate difference: 10.99–9.14 = 1.85. This means that the policy reduced the rate difference between lower and higher educational groups by almost 2 percentage points. Similarly, we calculated the policy effect on relative health inequality as the change of the rate ratio, which resulted in the finding that the policy has reduced the relative excess risk among the lower educational group by almost 12%.

## Discussion

### Summary of findings

This study demonstrated that all seven quantitative analytical methods identified can be used to quantify the equity impact of policies on absolute and relative inequalities in health by conducting an analysis stratified by socioeconomic position. Further, all but one (propensity score matching) can be used to quantify equity impacts by inclusion of an interaction term between socioeconomic position and policy exposure.

### Methodological considerations

In our example, we assessed the effects of the policy in stratified analysis, and modeled it by including an interaction term between policy exposure and education. Apart from our finding that an interaction term could not be included in propensity score matching and appeared to be slightly more complicated in instrumental variable analysis, some differences between both approaches have to be considered before deciding which approach to use. Stratification by education is intuitively attractive; the method, however, requires additional analyses to statistically test whether the policy effects for higher and lower socioeconomic groups differ. Comparing the overlap in confidence intervals provides some further insight, but is still not a formal test [33]. Further, in our simple example, we only had two levels for our indicator of socioeconomic position, which made stratification easy. Including more levels of socioeconomic position results in smaller strata, with loss of statistical power as a consequence. Moreover, some indicators of socioeconomic position can be measured on a continuous scale, such as number of years of education, or household income. Categorizing continuous values requires making (arbitrary) decisions, and results in a loss of information. Analyses using an interaction term allow indicators of socioeconomic position to be continuous variables. The results, however, can sometimes be more complex to interpret. For example, one issue to consider is that whether the effect of the policy on health inequalities changes in a linear way with an increase of one unit of the socioeconomic indicator.

Caution is needed when interpreting the results from the instrumental variable approach. Under certain conditions, the instrumental variable reveals a local average treatment effect [34], namely the intervention effect among individuals affected by the observed changes due to the instrument (“compliers”). It is a local parameter since it is specific to the population defined by the instrument [28]. Therefore, when the treatment effect is the same for everyone (homogenous treatment effect), all valid instruments will identify the same parameter. In this case, the local average treatment effect will be the same as the average treatment effect. However, in the more realistic cases with heterogeneous treatment effect, local average treatment effect is normally different from average treatment effect. Different instrumental variables, although all valid, will be associated with different local average treatment effect estimators and the population of corresponding compliers cannot be identified in the data [35]. Thus, when we apply it to health inequalities, for example in stratified analysis, the estimated policy effects are the effects among the corresponding compliers within each socioeconomic group given a set of instruments. The generalization of the conclusion to the whole population or to other populations is normally uncertain. However, when the change of policy is used as the instrument for the exposure, the local average treatment effect might be extremely useful, since it focuses on an important subpopulation whose exposure status is changed by the policy and may provide an informative measure of the impact of the policy [28].

The above mentioned problem of a low external validity also applies to regression discontinuity. Analysis are only performed based on the observations around a cutoff point (e.g. 1800–2200 euro per month in our example), and as a result, the method does not produce a policy effect on health inequalities among the whole population.

Persons were either exposed or unexposed in our fictitious example; we did not include the possibility of graded exposure to the policy. Whereas regression adjustment would allow a graded exposure relatively easy, for other techniques this may be more complex (although not impossible), such as for propensity score matching [36].

Which method to use depends to a large extent on data availability (e.g. whether cross-sectional or longitudinal data are available) and the nature of the confounders in the analysis (whether observable or not, and whether time-variant or not). The appropriateness of the preferred methods further depends on the degree to which underlying assumptions are met. For example, instrumental variable analysis requires strong assumptions, and violations can lead to biased estimated [21, 26]. Similarly, difference-in-differences analysis is based on the assumption of common trends between higher and lower educational groups in the absence of the policy.

### Interpretation

Although the methods described seem quite different, they actually try to achieve the same aim, which is constructing counterfactual outcomes for exposed units had they not been exposed to the policy [37]. Doing this in a convincing way is a key ingredient of any serious evaluation method [28]. For example, in well-designed randomized controlled trials, the control group is a perfect counterfactual for the exposed group, since the pre-intervention differences have been eliminated by the random assignment of intervention. In the same way, if the key assumption holds that selection bias disappears after controlling for observed characteristics [26], both regression adjustment and propensity score matching restore randomization to some extent. Similarly, both instrumental variable and regression discontinuity approaches aim at finding exogenous factors which can fully or partly determine the assignment to a policy; as such, this mimics randomization to some extent. If in a difference-in-differences analysis, the trend over time is the same for unexposed and exposed units of analysis, the change in the unexposed unit can be potentially used as the counterfactual. When longitudinal data is available, the fixed effects model uses the exposed unit’s own history prior to treatment to construct the counterfactual [20]. Likewise, the time trend of the exposed unit before the policy implementation is utilized as the counterfactual part when time-series data are available.

We constructed our fictitious data in the way that people with a low socioeconomic position were more exposed to the policy, but the policy effect among the exposed was equal between socioeconomic groups. In reality, health inequalities might also be reduced in cases where people with different socioeconomic positions are equally exposed to the policy, but where the policy effect among those exposed is larger among people with a low socioeconomic position. It can also happen that people with a low socioeconomic position are more exposed to the policy, and where the policy effect among those exposed is larger among people with a low socioeconomic position. The process of analysis and the interpretation of the results however, are similar for these cases. Moreover, although we mainly constructed the examples using individual level data (except for interrupted time-series), some methods can also be used with aggregated level data. For example, a fixed effects model can also be applied with country-level longitudinal data.

In this paper, we performed the analysis based on a standard setting of each method. The analysis however, can be easily extended to more complicated examples. We only used few covariates in our analysis, but more can be incorporated. It is also possible to use the methods with longitudinal or repeated cross-sectional data with multiple periods, continuous health outcomes, and more than one instrument. We used propensity score matching, as it is the most commonly used method of matching. However, other matching methods may be more effective in reducing imbalance, model dependence and bias, such as Mahalanobis Distance Matching and Coarsened Exact Matching [38]. Moreover, extensions of the models described here with relaxed assumptions have been applied, such as quasi difference-in-differences model [39], changes-in-changes model [40] and dynamic fixed effects model [41, 42]. The extended models are not covered by this paper, but the general way of applying them for assessing the impact of policy on health inequalities is similar to the standard models. Combining methods in one study is also possible. For example, some papers recommend to combine regression adjustment and propensity score matching by weighting the treated population by the inverse of the propensity score (i.e. the inverse probability weighting estimator) to reduce bias and improve efficiency [31, 43]. In this way, matching can be combined with many techniques such as difference-in-differences analysis and fixed effects model [44, 45]. Another example is incorporating instrumental variables into fixed effect models to tackle the potential measurement error [26].

This study demonstrated quantitative tools to assess if and to what extent natural policy experiments impact upon socioeconomic inequalities in health. While our approach offers further insight in whether effects resulted from a policy effects and/or and the size of the populations exposed, it does not offer in-depth insight into how effects were achieved. Quantitatively, (causal) mediation analyses could be used to assess explanations for potential effects, whereby the effect of the policy experiment on potential determinants could be assessed, as well as the effects of the potential explanatory factors on the outcome [46]. Future research should explore to what extent mediation analysis can be used to assess explanations of the impact of NPE’s in inequalities in health. Simultaneously, qualitative approaches can be used to further examine the processes leading to an impact [47, 48].

The demonstrated possibility to use the techniques described in this paper for studying the impact of NPE’s on socioeconomic inequalities raises the question as to whether all policy evaluations should include an evaluation of the equity impact. Researchers evaluating an equity impact of interventions are often criticized by statisticians for conducting unreliable (underpowered) analyses; those who don’t are at the same time however, criticized by policymakers in need of evidence what works to close the gap in health between socioeconomic groups [49]. Following guidelines in which a logic model includes theoretically plausible mechanisms for a reduction on inequalities in health, and in which statistical power is not a real issue, we recommend that an equity impact analyses should be an integral part of any policy experiment.

## Conclusions

In conclusion, application of methods commonly applied in economics and econometrics can be applied to assess the equity impact of natural policy experiments. The low external validity of results from instrumental variable and regression discontinuity makes these methods less desirable for assessing policy effects on population-level health inequalities. Increased use in social epidemiological research will help to build an evidence base to support policy making in this area.

## Footnotes

1 There was one disadvantage of using linear probability regressions with the fixed effects model in our dataset. Although the relative policy effect on health was independent of gender, there were some interaction effects between gender and policy if we use linear regression to assess the absolute policy effect. The absolute policy effect is lower among women, which is caused by a relatively lower prevalence of poor health among women. Strictly speaking, this makes the effect for women no longer “fixed” in an absolute setting. Women had a lower effect on health through the policy effect in the second period. The interaction effect however, was rather limited in our data, and did not lead to large discrepancies of the results from fixed effects models. Therefore, we decided to ignore these limited interaction effects between “female” and “policy”, and assumed that the variable “female” was still a “fixed effect” that can be eliminated by a fixed effects model.
